# The role of co-infections in *M. hyopneumoniae* outbreaks among heavy fattening pigs: a field study

**DOI:** 10.1186/s13567-022-01061-w

**Published:** 2022-06-13

**Authors:** Matteo Tonni, Nicoletta Formenti, M. Beatrice Boniotti, Flavia Guarneri, Federico Scali, Claudia Romeo, Paolo Pasquali, Maria Pieters, Dominiek Maes, Giovanni L. Alborali

**Affiliations:** 1 Istituto Zooprofilattico Sperimentale della Lombardia e dell’Emilia Romagna, Via Bianchi, 9, 25124 Brescia, Italy; 2 Department of Food Safety, Nutrition and Veterinary Public Health, Istituto Superiore di Sanità viale Regina Elena 299, 00161 Rome, Italy; 3 Department of Veterinary Population Medicine, University of Minnesota, 1365 Gortner Ave, St. Paul, MN 55108 USA; 4 Faculty of Veterinary Medicine, Ghent University, Merelbeke, Belgium

**Keywords:** Multiple-locus variable-number tandem repeat analysis, *Mycoplasma hyopneumoniae*, swine, variable-number tandem repeat type, fattening pigs

## Abstract

Little is known about how co-infections and genotype dynamics affect *Mycoplasma hyopneumoniae* infection in fattening pigs. This study was aimed at assessing the role of co-infections in *M. hyopneumoniae* outbreaks, their influence on the presence of *M. hyopneumoniae* genotypes and their impact on consequent lung lesions. Tracheobronchial swabs (TBS) from 300 finishers were collected from 10 farms at the onset of enzootic pneumonia outbreaks and 1 month later, sampling of 3 groups per farm: Group A showed clinical signs first, Group B was housed near Group A, and Group C was located in a different building. Pigs’ lungs were scored at the slaughterhouse. TBS were tested for the main pathogens involved in respiratory diseases, and samples positive for *M. hyopneumoniae* were genotyped by multiple-locus variable-number tandem repeat analysis (MLVA). Pigs in Group A showed the highest prevalence and load of *M. hyopneumoniae*. A positive association was detected between *M. hyopneumoniae* and *Mycoplasma hyorhinis*, whereas *Actinobacillus pleuropneumoniae* was more frequent when the *M. hyopneumoniae* load was higher. Nevertheless, co-infection had no effect on lung lesion scores. The presence of multiple MLVA types (mixed infections) increased in time only in pigs from Group C and was positively associated with porcine reproductive and respiratory syndrome virus infection. Lung lesions were more severe in pigs with at least one TBS positive for *M. hyopneumoniae* and in pigs with a history of mixed infections. The central role of *M. hyopneumoniae* and relevance of mixed infections suggest that increased biosecurity might be beneficial for lung lesion sequelae.

**Supplementary Information:**

The online version contains supplementary material available at 10.1186/s13567-022-01061-w.

## Introduction

*Mycoplasma hyopneumoniae* is one of the primary agents involved in porcine respiratory disease complex (PRDC) [1] along with a combination of other infectious viral and bacterial pathogens [1–4]. Multiple patterns of co-infection are possible [5] depending on the timing of infection and the pathogens involved. The immunomodulatory effect that viruses play in virus-bacteria superinfections is sometimes altered by *M. hyopneumoniae* that promotes both viral and bacterial infections. An example is the potentiation effect of *M. hyopneumoniae* during PRRSV co-infections that also leads to an increase in viral shedding [5]. The outcomes of co-infections vary depending on the type of interactions; i.e., synergy, neutrality, or antagonism among the microorganisms [5]. The clinical signs and lung lesions of *M. hyopneumoniae* infections depend on many factors [2], such as viral and bacterial superinfections [1] or the presence of different circulating variable-number tandem repeat (VNTR) types. The effects of lung microbiota on lung health [6] and possible lung lesion sequelae are still unclear, although some studies are starting to shed light on this topic [7, 8]. *M. hyopneumoniae* is one the most common agents associated with pneumonia in the slaughterhouse setting [5, 9]. The impact of other co-infecting microorganisms on lung lesions has been hypothesized [10] and investigated by experimental infection [11, 12], but studies performed under field conditions are lacking. The genomic variability of *M. hyopneumoniae* [13–17] and the resulting lung lesions [18] have been widely studied. However, little is known about the role of co-infections on *M. hyopneumoniae* genetic diversity and their differential effects on the resulting lung sequelae.

This study was performed to assess the dynamics of *M. hyopneumoniae* infection during clinical respiratory outbreaks in heavy fattening pigs. In particular, the main purpose was to clarify the role of co-infections in *M. hyopneumoniae* outbreaks and their impact on consequent lung lesions. An additional aim was to investigate how co-infection affects the presence of *M. hyopneumoniae* genotypes and whether infections of multiple VNTR types might influence the magnitude of lung lesions.

In this study, *M. hyopneumoniae* seemed to play a central role in respiratory outbreaks in fattening pigs, with a direct effect on lung lesion scores. Moreover, the presence of *M. hyopneumoniae* infections of multiple VNTR types led to more severe lung lesions. Otherwise, co-infections were found to have marginal effects with no relevant influence on the severity of lung lesions.

## Materials and methods

### Study design

This study was performed from May 2016 to April 2018 in 10 fattening farms. All the enrolled farms belonged to multisite production flows with a history of *M. hyopneumonaie.* All pigs enter the fattening units at the approximate age of 60 days. Veterinary practitioners belonging to two major practices were asked to immediately contact Istituto Zooprofilattico Sperimentale della Lombardia e dell’Emilia Romagna (IZSLER) if they suspected an enzootic pneumonia (EP) outbreak in finishing pigs. Herds in which fattening pigs showed continuative clinical signs of sneezing and a dry non-productive cough [19] were enrolled in the present study. Herds in which pigs showed signs such as fever, anorexia, and laboured breathing were excluded because these signs suppose the presence of other bacterial and/or viral agent outbreaks [19].

In every farm, 3 groups (Groups A, B, and C) of 10 pigs each were defined in relation to the animals that manifested symptoms first. Pigs within each group were randomly selected. Group A comprised pigs that first showed acute clinical signs. Group B comprised asymptomatic pigs housed in the pen next to Group A. Group C comprised asymptomatic pigs housed in another barn of the farm. The pigs enrolled in the study were individually identified with ear tags. The housing conditions were the same for Groups A, B, and C. Tracheobronchial swabs (TBS) were simultaneously collected from all 30 pigs on each farm at two time points: at the onset of clinical signs in Group A (T0) and 32 ± 6 days later, a reasonable time interval for *M. hyopneumoniae* infection to turn into the recovery phase (T1) [1]. Lungs of the selected pigs were scored for lesions at the slaughterhouse (T2).

### Farm management

All farms were located in the northern region of Italy and followed an all-in/all-out protocol within a multisite production flow system. Pigs were housed in confined barns with natural ventilation systems. Vaccination protocols included Aujeszky disease virus, porcine circovirus type 2 (PCV2), and *M. hyopneumoniae*. On all farms, vaccination against Aujeszky disease was performed three times (the last during the fattening phase), whereas PCV2 vaccination involved a single shot in the farrowing site. The *M. hyopneumoniae* vaccination strategies are shown in Table 1. None of the considered fattening farms adopted the anthelmintic treatment regime.

**Table 1:** **Description of the ten fattening pig herds included in the study**

Farm	Herd Size (Nb of pigs)	Enrolled pigs	*M. hyopneumoniae* vaccination schemes*	Age of pigs at T0 (days)	Age at slaughtering
1	4000	30	1	131	245
2	4800	30	1	153	250
3	10 000	30	1	176	270
4	1500	30	1	204	280
5	3200	30	1	158	255
6	2300	30	2	238	265
7	3900	30	2	143	280
8	5300	30	2	232	275
9	1500	30	1	206	265
10	4500	30	2	124	255

^*^ Vaccination strategies were resumed in 1 (single shot at 20–28 days) or 2 (double shots before and after weaning).

T0: onset of clinical signs during the EP outbreak.

None of the selected pigs had undergone any pharmacological treatment with the exception of individual therapy with tetracyclines or macrolides administered to animals not under study in all farms following the onset of respiratory symptoms.

### Sampling procedures

Tracheobronchial swabs (TBS) were collected through a 60-cm catheter (Portex® Catheter; Smiths Medical, Minneapolis, MN, USA). As the pig inspired, the catheter was inserted into the trachea with the aid of a laryngoscope and mouth speculum. The catheter was gently inserted deeply into the trachea until the first branching of the bronchial tree in accordance with a standard procedure [20]. Immediately after sampling, the tip of the catheter was inserted into a sterile tube with 2 mL of phosphate-buffered saline (PBS) and transferred under refrigerated conditions (5 °C ± 3 °C) to the IZSLER laboratory.

Lungs were scored after slaughter following the method proposed by Madec and Kobisch [21] based on the magnitude of greyish to purplish consolidated pneumonic areas on each lung lobe surface: 0% = no lesion, <25% = score of 1, 25%–49% = score of 2, 50%–74% = score of 3, and >75% = score of 4. Based on the total lung score (range of 0 to 28), the severity of lung lesions was classified as suggested by Hillen et al. [22]: no lesions (score of 0), mild lesions (score of 1–4), severe lesions (score of 5–9), or very severe lesions (score of ≥ 10).

### Pathogen detection

Pathogen investigation focused on *M. hyopneumoniae* and the main PRDC agents known to occur in the considered geographic area [4]: *Actinobacillus pleuropneumoniae*, *Mycoplasma hyorhinis (M. hyorhinis)*, *Glässerella parasuis*, *Bordetella bronchiseptica*, *Trueperella pyogenes*, porcine reproductive and respiratory syndrome virus (PRRSV), PCV2, swine influenza A virus (swIAV), and *Pasteurella multocida* [4]. Samples positive for *M. hyopneumoniae* were also genotyped by multiple-locus variable-number tandem repeat analysis (MLVA).

#### Bacterial isolation

Because of its ease and reliability of execution [1], bacterial culture was adopted to detect *P. multocida*, *B. bronchiseptica*, and *T. pyogenes.* A drop of the tracheobronchial secretions contained in the sterile tube with TBS and PBS was seeded onto a blood agar culture plate through a sterile loop. All plates were incubated at 37 °C in an aerobic atmosphere for 24 ± 3 h. The bacterial growth was evaluated by examining the morphology of the colony, performing Gram staining, and performing biochemical tests.

In the case of a mucous colony, *P. multocida* was suspected and specific biochemical tests (API NE; bioMeriéux) were carried out to confirm the pathogen identity [23]. No colony suspected to be *B. bronchiseptica* or *T. pyogenes* was detected; therefore, these two pathogens were excluded from the study.

Tracheobronchial swab samples were considered positive when at least one colony of *P. multocida* was detected.

#### Molecular biology

The biomolecular assays used in the present study were chosen to meet the requirements of the quality management system of IZSLER based on UNI CEI EN ISO/IEC 17025 [24].

DNA and RNA were extracted from TBS and lung homogenates using an automated extraction platform (KingFisher™; Thermo Fisher Scientific, Waltham, MA, USA). The eluted nucleic acids then underwent molecular tests. Real-time polymerase chain reaction (PCR) for *M. hyopneumoniae*, *M. hyorhinis*, *A. pleuropneumoniae*, PRRSV, PCV2, and swIAV were carried out using a C1000 thermal cycler (Bio-Rad Laboratories, Hercules, CA, USA), while conventional PCR (*G. parasuis*) was performed on an Applied Biosystems GeneAmp® PCR System 9700 (Thermo Fisher Scientific).

The real-time PCR adopted to detect *M. hyopneumoniae* followed the protocol proposed by Marois et al. [25] with the detection limit set at a 37-cycle threshold (Ct) value. Positive samples were further submitted to MLVA genotypic analysis.

Another real-time PCR assay based on the gene encoding *M. hyorhinis* protein p37 [26] was developed to detect this mycoplasma, setting a 38-Ct limit. PRRSV RNA was detected using the Applied Biosystems LSI VetMAX™ PRRSV EU/NA real-time PCR kit (Thermo Fisher Scientific) as specified by the manufacturer, with a threshold detection limit of 37-Ct. The presence and quantification of PCV2 was assessed through real-time PCR according to the protocol published by Olvera et al. [27]; the IZSLER laboratory threshold limit was set at 40-Ct. Because of the potentially low sensitivity of TBS for PRRSV and PCV2, serology data was collected at both T0 and T1, to better assess the farm status, resulting in seroprevalences > 90% for both cases and at both time points. SwIAV was detected through real-time reverse-transcriptase PCR with amplification of the M gene of influenza A virus following the protocol described in the Manual of Diagnostic Tests and Vaccines for Terrestrial Animals [28]. Detection of *G. parasuis* was performed using the qualitative PCR protocol standardized by Oliveira et al. [29]; the PCR product was loaded on a 2.0% agarose gel, run at 100 V for 40 min, and visualized under ultraviolet light.

### *Mycoplasma hyopneumoniae* MLVA genotyping

For the samples positive for *M. hyopneumoniae* by real-time PCR, the number of copies was elaborated based on a standard reference curve. These samples were genotyped after with MLVA. Briefly, the method consisted of four conventional PCR for the amplification of Locus 1, Locus 2, P97-RR1, and P97-RR2 genes in accordance with the scheme proposed by Charlebois et al. [13] and Tonni et al. [17]. The PCR were performed with an Applied Biosystems thermocycler (Thermo Fisher Scientific), and the PCR products were examined by capillary electrophoresis (QIAxcel; Qiagen, Hilden, Germany). In case of unclear results, further evaluation was performed with a 2.0% high-resolution agarose gel run at 100 V for 2 h and visualized under ultraviolet light. For each VNTR locus, the estimated number of tandem repeats was calculated according to the allele calling table (Additional file 1). Each VNTR type was defined based on the number of repeats per locus. The VNTR type nomenclature was assigned following the chronological order of identification at the IZSLER laboratory.

The presence of one or more *M. hyopneumoniae* VNTR types per sample was defined as a single (SN) or mixed (MX) infection, respectively.

### Statistical analysis

For all 300 pigs, the probability of being infected by *M. hyopneumoniae* during the outbreak and before slaughtering was examined through a mixed logistic regression, including time (T0 or T1), experimental group (A, B, or C), time × group interaction, and infection status (infected or not infected by other pathogens) as explanatory variables. In the subset of pigs infected by *M. hyopneumoniae* (*n* = 229), we also explored the variation in the ln-transformed number of copies through a general linear mixed model, including the same set of explanatory variables. Finally, the effect of these same variables on the probability of having an MX infection was analysed through a second mixed logistic regression. In all these models, pig and farm ID were included as random factors to account for repeated measures on the same individual and for within-farm variability, respectively. In all cases, models in which the farm was included as a random intercept were significantly different from simple models (all *p* < 0.0001).

Finally, we explored variation in the aggregated lung lesion scores of pigs (i.e., no lesions, mild lesions, severe lesions, or very severe lesions) observed at T2 through two different mixed ordinal logistic regressions. In the first model, we included all pigs (*n* = 300) and examined the effect of the group and *M. hyopneumoniae* infection status (i.e., infected at least once in time or never infected). In this model, we also included the infection status by *M. hyorhinis*, *A. pleuropneumoniae* and *P. multocida*, because they are known to potentially cause severe lung lesions. In the second model, we included only animals that had been infected at least once by *M. hyopneumoniae* (*n* = 280) and considered as explanatory variables the experimental group, the total number of co-infections, and MLVA history (i.e., MX genotype infection at least once in time or always SN infection). In both models, the farm ID was included as a random intercept.

In all analyses, we started from full models and obtained minimal models through backward elimination of non-significant variables (partial *p*-value for removal set at 0.15). For the linear model, comparisons of significant variables with more than two levels were analysed through t-tests on differences in least squares means, applying Holm correction for multiple comparisons. For logistic regressions, odds ratio (OR) estimates and their 95% confidence intervals (CIs) are reported. For the linear mixed model, normality of residuals was assessed visually. For logistic regressions, overdispersion was checked through the generalized chi-square/degree of freedom ratio, and the areas under the receiver operating characteristic curves (AUC) are reported below.

All analyses were carried out through PROC GLIMMIX in SAS/STAT 9.4 software (SAS Institute Inc., Cary, NC, USA).

## Results

### *Mycoplasma hyopneumoniae* infection and VNTR types

Overall, 442 of the 586 examined TBS (corresponding to 300 different pigs) were positive for *M. hyopneumoniae* (75.4%; 95% CI 71.9–78.9). The prevalence of positive swabs in farms ranged from 8.5% to 100%. A detailed breakdown of the prevalence of *M. hyopneumoniae* and all other infections by time and by experimental group is provided in Table 2. From T0 to T1, 11 pigs of Group C that developed non-respiratory disease and 3 pigs (1 in each group) that died were excluded from the study.

**Table 2:** **Respiratory infections in heavy-fattening pigs: prevalence (% of infected animals/examined animals) at T0 and T1 and incidence (% of infected animals/susceptible animals) at T1 in Group A (N**_**T0**_** = 100, N**_**T1**_ **= 99), Group B (N**_**T0**_** = 100, N**_**T1**_ **= 99) and Group C (N**_**T0**_ **= 100, N**_**T1**_** = 88)**

Infection	Group	T0		T1			
		P (%)	95% CI (%)	P (%)	95% CI (%)	I (%)	95% CI (%)
*M. hyopneumoniae*	A	85.0	77.9–92.1	86.9	80.1–93.6	26.7	4.3–49.0
	B	82.0	74.3–89.7	73.7	64.9–82.6	27.8	7.1–48.5
	C	62.0	52.3–71.7	61.4	51.0–71.7	29.7	15.0–44.5
*A. pleuropneumoniae*	A	27.0	18.1–35.8	24.2	15.6–32.8	23.3	13.6–33.0
	B	19.0	11.2–26.8	20.2	12.1–28.2	22.2	13.2–31.3
	C	22.0	13.7–30.3	14.8	7.2–22.3	12.3	4.8–19.9
*G. parasuis*	A	40.0	30.2–49.8	55.6	45.6–65.5	50.8	38.1–63.6
	B	28.0	19.0–36.9	56.6	46.6–66.5	52.1	40.5–63.7
	C	31.0	21.8–40.2	40.9	30.4–51.4	31.7	19.9–43.4
*M. hyorhinis*	A	67.0	57.6–76.4	64.6	55.1–74.2	59.4	42.4–76.4
	B	64.0	54.4–73.6	59.6	49.7–69.4	54.3	37.8–70.8
	C	55.0	45.1–64.9	45.4	34.8–56.1	35.7	21.2–50.2
*P. multocida*	A	52.0	42.0–62.0	8.1	2.6–13.5	4.2	0–9.8
	B	7.0	1.9–12.1	36.4	26.7–46.0	35.9	26.1–45.7
	C	5.0	0.6–9.3	22.7	13.8–31.6	22.9	13.8–31.9
PCV2	A	11.0	4.8–17.2	8.1	2.6–13.5	0	–
	B	9.0	3.3–14.7	7.1	1.9–12.2	0	–
	C	8.0	2.6–13.4	10.2	3.8–16.7	1.7	0–5.9
PRRSV	A	11.0	4.8–17.2	2.0	0–4.8	1.1	0–3.3
	B	6.0	1.3–10.7	1.0	0–3.0	1.0	0–3.2
	C	3.0	0–6.4	0	0	0	–
SwIAV	A	0	–	0	–	0	–
	B	0	–	0	–	0	–
	C	2.0	0–4.8	0	–	0	–

**Figure 1:**
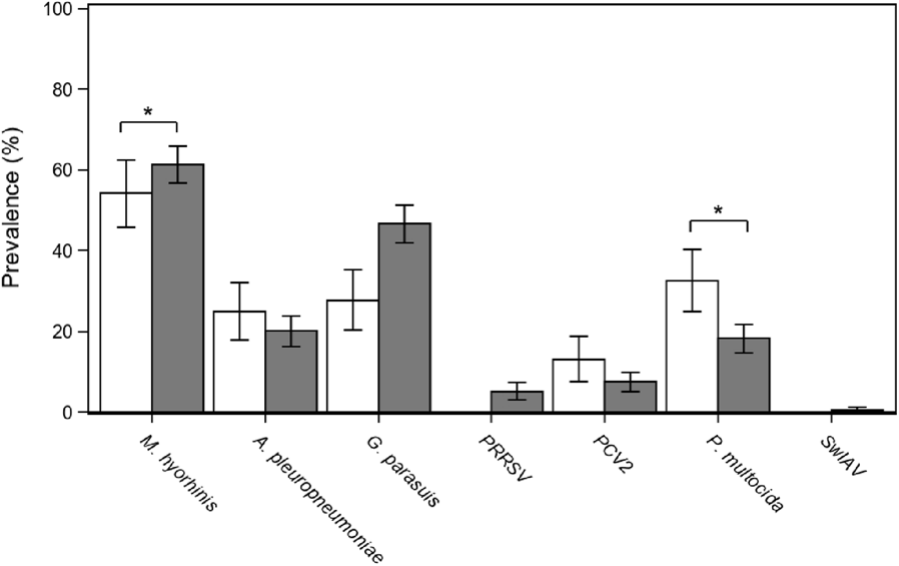
***M. hyopneumoniae* co-infections in fattening pigs.** Prevalence of other respiratory infections in pigs infected (dark bars, *n* = 442) and not infected (white bars, *n* = 144) by *M. hyopneumoniae.* Bars indicate 95% Confidence Intervals. Asterisks indicate significance level at *p* < 0.05 in the mixed logistic regression model.

The probability of a pig being infected by *M. hyopneumoniae* varied significantly with groups, by the *M. hyorhinis* infection status, and by the *P. multocida* infection status (all *p* < 0.05) (mixed logistic regression, *N* = 600 samples, AUC = 0.93; Table 3). In detail, pigs from Group A had higher odds of being infected by *M. hyopneumoniae* than pigs from both Group B (OR, 2.7; 95% CI 1.1–6.2) and Group C (OR, 11.7; 95% CI 4.9–27.9), whereas pigs from Group B were more likely to be infected than pigs from Group C (OR, 4.4; 95% CI 2.1–9.3). Additionally, co-infection by *M. hyorhinis* was positively associated with the *M. hyopneumoniae* infection status (OR, 1.8; 95% CI 1.1–3.0), whereas *P. multocida* infection was instead negatively associated with it (OR, 0.5; 95% CI 0.6–0.9). Overall, the prevalence of *M. hyorhinis* was indeed higher in pigs co-infected by *M. hyopneumoniae*, whereas the prevalence of *P. multocida* was conversely higher in pigs free from *M. hyopneumoniae* infection (Figure 1).

**Table 3:** **Minimal models exploring factors affecting variation in** ***M. hyopneumoniae*** **infection status, ln-transformed no. of copies and VNTR types in pigs**

Response variable	Explanatory variable		Parameter estimate ± SE	Statistic	*p*-value
*M. hyopneumoniae* infection status^†^	Group^b^	A	2.67 ± 0.55	χ^2^_2_ = 32.8	**<0.0001**
		B	1.98 ± 0.47		
	Time^c^	T_1_	0.37 ± 0.33	χ^2^_1_ = 0.25	0.62
	*M. hyorhinis* infection status^d^	Infected	0.58 ± 0.27	χ^2^_1_ = 4.59	**0.032**
	*P. multocida* infection status^d^	Infected	−0.71 ± 0.33	χ^2^_1_ = 4.49	**0.034**
	Group:Time^b, c^	Group A: T_1_	−0.42 ± 0.61	χ^2^_2_ = 4.01	0.13
		Group B: T_1_	−1.01 ± 0.50		
*M. hyopneumoniae* no. of copies (ln-transformed)^*, a^	Group^b^	A	3.45 ± 0.54	F_2, 425_ = 19.7	**<0.0001**
		B	2.04 ± 0.55		
	Time^c^	T_1_	0.03 ± 0.59	F_1, 425_ = 14.7	**0.0001**
	*M. hyorhinis* infection status^d^	Infected	1.11 ± 0.33	F_1, 425_ = 11.2	**0.0009**
	*A. pleuropneumoniae* infection status^d^	Infected	0.65 ± 0.40	F_1, 425_ = 2.70	0.10
	Group:Time^b, c^	Group A: T_1_	−1.84 ± 0.75	F_2, 425_ = 3.48	**0.032**
		Group B: T_1_	−1.74 ± 0.77		
*M. hyopneumoniae* MLVA^†, a^	Group^b^	A	−1.73 ± 0.51	χ^2^_2_ = 1.02	0.60
		B	−1.18 ± 0.49		
	Time^c^	T_1_	2.41 ± 0.59	χ^2^_1_ = 9.20	**0.0024**
	PRRSV infection status^d^	Infected	1.13 ± 0.56	χ^2^_1_ = 4.09	**0.043**
	Group:Time^b, c^	Group A: T_1_	−2.54 ± 0.70	χ^2^_2_ = 13.42	**0.0012**
		Group B: T_1_	−2.13 ± 0.71		

In all the models, pig IDs and farm IDs were included as random intercepts.

^†^mixed logistic regression; ^*^ linear mixed model; ^a^ only positive animals; ^b^ Group C held as reference; ^c^ T0 held as reference; ^d^ not-infected held as reference.

The number of copies of *M. hyopneumoniae* in positive pigs ranged from 7.1 to 21.8 ln-copies [mean ± standard error (SE), 14.8 ± 0.2]. It increased with both *M. hyorhinis* (parameter estimate ± SE, 1.11 ± 0.33 ln copies) and *A. pleuropneumoniae* co-infection (0.65 ± 0.40 ln copies) and varied significantly in time depending on the group (all *p* < 0.05) (linear mixed model, *N* = 442 samples; Table 4). At T0, there was no difference in the number of copies between positive animals from Groups A and B (p_adj_ = 0.055), but they both had a significantly higher number of copies than positive pigs from Group C (p_adj_ < 0.0001 and *p* = 0.003, respectively). However, the number of copies in Groups A and B decreased significantly from T0 to T1 (p_adj_ = 0.002 and p_adj_ = 0.01), while the average number of copies in positive pigs from Group C did not change over time (p_adj_ = 0.95). As a consequence, at T1 there was no longer a difference in the number of copies among any of the groups (all p_adj_ > 0.05). Detailed results of the DSLM post-hoc comparisons are shown in Table 4.

**Figure 2:**
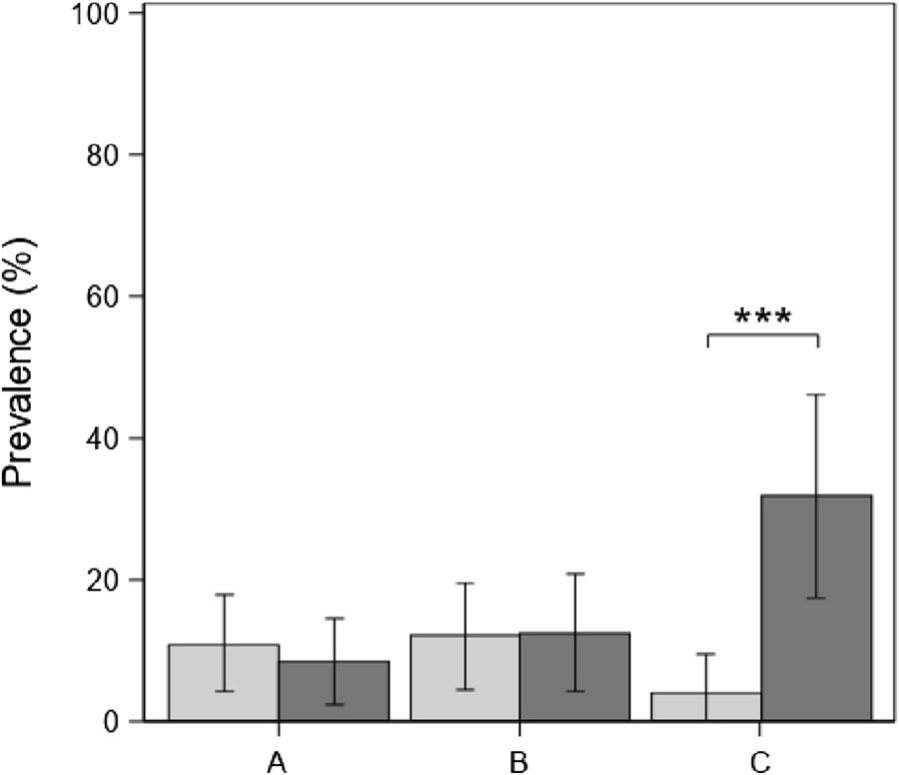
**Dynamics of *M. hyopneumoniae* mixed infections in fattening pigs.** Prevalence of *M. hyopneumoniae* mixed infections (i.e. showing multiple VNTR genotypes) in positive pigs at T0 (light bars) and T1 (dark bars) per experimental group. Error bars indicate 95% Confidence Intervals. Asterisks indicate significance level at p_adj_ < 0.001 in t-tests on differences of least squares means.

With respect to the VNTR types, the overall prevalence of MX infections in the examined positive samples was 12.3% (95% CI 9.0–15.5). A breakdown of prevalence by time and experimental group is shown in Table 3. The probability of positive pigs having an MX infection increased significantly with PRRSV co-infection (OR, 3.1; 95% CI 1.1–9.3) and varied in time depending on the group (both *p* < 0.05) (mixed logistic regression, N = 400 samples; AUC = 0.84; Table 3). In detail, in Groups A and B there was no variation in the occurrence of MX infection from T0 to T1, whereas in pigs from Group C there was a significant increase in the probability of showing an MX at T1 (OR, 11.1; 95% CI 3.5–36.5; p_adj_ = 0.0008) (see also Figure 2).

**Table 4:** **Differences of least square means by time and group in** ***M. hyopneumoniae*** **no. of copies (ln-transformed) in samples (*N*** **= 442) from infected pigs**

Time	Group	Estimate ± SE	t_425_	p_adj_
T_0_	A vs B	1.41 ± 0.47	2.83	0.055
	A vs C	3.45 ± 0.54	6.37	**<0.0001**
	B vs C	2.04 ± 0.55	3.72	**0.0031**
T_1_	A vs B	1.31 ± 0.51	2.54	0.11
	A vs C	1.61 ± 0.57	2.82	0.056
	B vs C	0.30 ± 0.58	0.51	0.99
T_1_ vs T_0_	A	−1.81 ± 0.47	−3.84	**0.0020**
	B	−1.71 ± 0.50	−3.40	**0.010**
	C	0.03 ± 0.59	0.06	0.95

### Lung lesions

The raw total lung lesion scores in pigs examined at T2 (*n* = 287) ranged from 0 to 22 (mean ± SE, 4.9 ± 0.3). Ninety-two pigs showed no lesions, 76 pigs showed mild lesions, 67 pigs showed severe lesions, and 52 pigs showed very severe lesions as shown in Table 5. Overall, the probability of observing severe lesions was significantly affected only by a history of *M. hyopneumoniae* infection (χ^2^_1_ = 20.15; *p* < 0.0001), with pigs infected at least once having 40 times the odds of showing very severe lesions. Indeed, of the 19 animals that were never infected by *M. hyopneumoniae*, 17 showed no lesions and 2 showed mild lesions; none showed severe or very severe scores. Neither the experimental group nor any of the other respiratory infections included in the model had a significant effect on lung lesions (all *p* > 0.05). Considering only pigs with a history of *M. hyopneumoniae* infection, lung lesions were affected only by *M. hyopneumoniae* (χ^2^_1_ = 12.1; *p* < 0.0001). Pigs with MX infections had significantly higher odds of showing severe lesion scores than pigs with SN infections (OR, 3.0; 95% CI 1.6–5.6). Once again, neither the experimental group nor the number of co-infections had any effect on the severity of lung lesions (*p* = 0.36 and *p* = 0.76, respectively).

**Table 5:** **Severity of lesions based on the lung lesion score according to the Madec and Kobish method [**18**]**

Magnitude of lesion	P (%)	95% CI (%)
No lesion^a^	32.1	26.6–37.5
Mild lesions^b^	26.0	21.3–31.6
Severe lesions^c^	23.3	18.4–8.3
Very severe lesions^d^	18.1	13.6–22.6

Data refer to all 10 farms for a total of 287 scored pigs.

^a^Score = 0; ^b^ score 1–4; ^c^ score 5–9; ^d^ score ≥ 10.

## Discussion

The present study was performed to investigate the dynamics of *M. hyopneumoniae* and its interactions with other respiratory pathogens in pig farms. The farms enrolled in the study were therefore specifically selected because of reported clinical signs such as non-productive coughing and sneezing, which led to the suspicion of EP outbreaks. Consequently, the prevalence of *M. hyopneumoniae* in our sampled population was indeed higher than that of all other respiratory infections.

The three different groups of pigs sampled in each farm were defined according to farm localization and clinical conditions, leading to different exposures to *M. hyopneumoniae* among the groups independent of the sampling time. Group A included pigs in which clinical signs were first observed, and as expected, this group had the highest probability of being infected by *M. hyopneumoniae*. Conversely, animals housed near Group A or stalled in another building (Group B and C, respectively) were less likely to be positive for *M. hyopneumoniae*.

On the contrary to the infection status, the *M. hyopneumoniae* load varied in time but with a different pattern depending on the group. The highest PCR number of copies was detected at T0 in Groups A and B and at T1 in Group C. Because the highest burden of *M. hyopneumoniae* has been proven to occur at 28 days post-infection [30], it can be assumed that the early stages of the EP outbreaks occurred at T0 in Groups A and B and at T1 in Group C. Considering the higher sensitivity of real-time PCR on TBS samples collected in the early stage of the outbreak [31], these findings suggest that sampling of pigs with clinical signs may lead to a more effective and timely diagnosis of EP outbreaks.

The role of *M. hyopneumoniae* as a gate opener for secondary bacterial infections, especially *P. multocida* and *A. pleuropneumoniae*, is well known [2, 19]. In the present study, the wide panel of analysed pathogens allowed for the study of several possible co-infections, but the only positive association was detected between *M. hyopneumoniae* and *M. hyorhinis*, with the latter more frequently observed in the presence of the former. *Mycoplasma hyopneumoniae* is one of the main pathogens involved in swine pneumonia [1], whereas *M. hyorhinis* generally has slight importance with mild or no clinical respiratory signs [32]. No positive association was detected between the presence of *M. hyopneumoniae* and other respiratory pathogens, with *P. multocida* even showing a slightly negative association with *M. hyopneumoniae* infection. The prevalence of *P. multocida* was indeed higher in pigs free from *M. hyopneumoniae*, which may suggest an unfavourable interaction between these two bacterial infections. However, a possible explanation for this observation is the strict selection criteria of the enrolled farms, which included pigs with signs of EP but without the typical signs of a complicated infection (e.g., pyrexia, anorexia, lethargy, or even death) [1]. In other words, the rigorous enrolment of farms with pigs showing typical signs of EP may have promoted the selection of pure and uncomplicated *M. hyopneumoniae* infections; therefore, the presence of *P. multocida* was presumably not due to secondary irruption. Furthermore, because *P. multocida* was isolated on only a few farms, we cannot exclude the possibility that this weak negative association was due to the limited sample size.

Although no association was detected between the presence of *M. hyopneumoniae* and *A. pleuropneumoniae*, co-infection by these two bacteria was found to be more common when *M. hyopneumoniae* showed a higher number of copies in the PCR assay. Similarly, infections with *M. hyorhinis* were more frequent with a higher *M. hyopneumoniae* load. Despite the fact that the exact mechanisms of co-infections in respiratory diseases are not well known [5], a possible explanation for this result is the need for *M. hyopneumoniae* to be well established in the respiratory tract to potentiate secondary pathogens [5]. Consequently, containing the proliferation of *M. hyopneumoniae* could be beneficial to avoid *A. pleuropneumoniae* and *M. hyorhinis* co-infections.

The prevalence of several respiratory pathogens seemed to vary with time and by group, but the often-limited number of infected pigs coupled with the non-random selection of farms did not allow for a more detailed analysis of the other infection dynamics. Furthermore, the vaccination strategies (including the type of vaccine used) were not considered as variables in this study and should be investigated in the future.

The respiratory pathogens considered in this study are the most common agents detected in pig farms in the study area [4]; therefore, since no deworming scheme has been adopted and because *Ascaris suum* was not considered, this should be considered a limitation of the research.

*Actinobacillus pleuropneumoniae* is a major agent of chronic pleurisy [1]. However, we rarely found pleural lesions in our sample, and this prompted us to not consider the pleurisy score.

Concerning the dynamics of *M. hyopneumoniae* VNTR types, the prevalence of *M. hyopneumoniae* MX infections was stable through time in Groups A and B, whereas it increased significantly from T0 to T1 in Group C. Considering the aforementioned trend of the *M. hyopneumoniae* load that suggested the onset of the outbreak at T0 in Groups A and B and at T1 in Group C, the MX infections appeared to occur mainly in pigs later and farthest from where the outbreak of EP started.

Since the endemic presence of *M. hyopneumoniae* complicates the interpretation of these findings, the currently available data did not allow us to assess the actual mechanism that is occurring. However, a possible interpretation is that when an EP outbreak occurs, the VNTR types involved remain the same throughout the pathogenic evolution within each pig. The subsequent phase of the outbreak with extensive shedding of *M. hyopneumoniae* instead induces the circulation of high loads of different VNTR types, increasing the risk of pigs being exposed to multiple VNTR types. The data show a complex dynamic of different genotype infections with an evolution of the VNTR types along the different stages of the outbreak. Therefore, the timing of sampling could greatly affect the likelihood of detecting all genotypes hosted by a pig. However, these results should be interpreted with caution because although TBS are considered a very sensitive type of sample for *intra vitam* diagnosis [20], VNTR types are individually hosted in each lung lobe [17], and the ability of TBS to detect all VNTR types in each lung has never been assessed.

TBS analysis was our primary choice because it is the most sensitive method for the diagnosis of *M. hyopneumoniae* in live animals [20]. However, its sensitivity for some of the other investigated respiratory pathogens is either lower or unknown. For instance, *A. pleuropneumoniae* and *G. parasuis* are more reliably cultured from lung tissue [1, 33], and this should be considered a partial limitation of this procedure. Despite this, TBS were chosen because they would have allowed for the successful detection of the widest range of pathogens among those known to circulate in the study area.

Additionally, we found that the probability of *M. hyopneumoniae*-positive pigs having an MX infection increased significantly with PRRSV co-infection. The presence of active PRRSV circulation is considered an indirect index of biosecurity [34], and low biosecurity has also been suspected of being related to infection with multiple *M. hyopneumoniae* VNTR types [35]. Thus, improvement in external biosecurity could prevent the entrance of pathogens such as PRRSV [34] and might also avoid the introduction of several *M. hyopneumoniae* genotypes. However, these results should be interpreted with caution because of the very limited presence of PRRSV in our sample, and further investigations are surely needed to draw more substantial conclusions.

The high prevalence of *M. hyopneumoniae* among lung lesion-associated bacteria is well known [5], and in the present study we indeed observed significantly more severe lung lesions in pigs with at least one positive PCR for *M. hyopneumoniae*. However, the severity of lesions was not related to the group, suggesting that the time point at which pigs become infected during the outbreak is not important; the critical point is rather the infection by *M. hyopneumoniae* itself. A practical implication of this is that in vivo samplings during an EP outbreak could allow for early identification of pigs that will likely develop lung sequelae.

Studying co-infections is very complex and challenging, and the evolution of co-infections in pigs during the fattening period is a largely unexplored field [5]. The present study partially sheds light on *M. hyopneumoniae* and other respiratory co-infections during EP outbreaks. Previous studies have revealed the ability of *M. hyorhinis* to exacerbate *M. hyopneumoniae* lung lesions [32]. In the present study, the presence and load of *M. hyopneumoniae* in TBS were significantly associated with the presence of *M. hyorhinis*, but this co-infection was not associated with a worsening of lesion severity. Furthermore, despite the well-known ability of *A. pleuropneumoniae* and *P. multocida* to cause lung lesions [36], no relationship has been detected between the presence of these two bacteria and the magnitude of lesions. In general, the presence of multiple co-infections was not related to the severity of lung lesions in our sample, indicating that *M. hyopneumoniae* has a dominant role in determining their magnitude*.*

The longer finishing period adopted in Italy surely influences the dynamics of infection [1]. Indeed, an extensive study on Italian pig farms revealed that a history of *A. pleuropneumoniae* infection is a risk factor for pleuritis sequelae [37]. Likewise, *M. hyopneumoniae*, which has the same tendency to become chronic, could follow a similar evolution. Notably, however, the clinical evaluation of pigs in the present study was carried out only at the beginning of the outbreak (T0) and after slaughtering (T2) by lung lesion scoring. An additional evaluation of the clinical status at T1 could indeed help to shed further light on the outbreak evolution.

The relationship between the detection of specific strains of *M. hyopneumoniae* or the presence of multiple VNTR types and lung lesion scores is controversial [13, 17, 18, 38, 39]. In this study, pigs with a history of MX infection during the outbreak show more severe lung lesions. Therefore, an improvement in lung lesion scores could be achieved by limiting the circulation of diverse genotypes (e.g., by improving farm biosecurity as mentioned above), as suggested by Michiels et al. [18].

In conclusion, *M. hyopneumoniae* was found to play a central role in respiratory outbreaks in fattening pigs, while co-infections seemed to play a more marginal role with no relevant effect on the severity of lung lesions. The presence of *M. hyopneumoniae* infections of multiple VNTR types during the finishing period led to more severe lung lesions in this study. Therefore, improving farm management and biosecurity and in turn limiting the circulation of multiple genotypes could potentially lead to a better lung lesion status.

## Supplementary Information

**Additional file 1.** **Allele calling table.** Each VNTR type is identified as a combination of different VNTR locus. The number of repeats of each locus is determined by the number of base pairs (bp) for that locus.

## Data Availability

The datasets used and/or analysed during the current study are available from the corresponding author upon reasonable request. The dataset supporting the conclusions of this article is included within the article.
