# Supplementary material for: The role of co-infections in M. hyopneumoniae outbreaks among heavy fattening pigs: a field study
Source: Vet Res. 2022 Jun 13;53:41. doi: 10.1186/s13567-022-01061-w (PMC9190078; doi:10.1186/s13567-022-01061-w)
Supplement: Supplementary file 1 — Additional file 1. Allele calling table. Each VNTR type is identified as a combination of different VNTR locus. The number of repeats of each locus is determined by the number of base pairs (bp) for that locus. [file 13567_2022_1061_MOESM1_ESM.docx]

|  | Locus 1 | |  | Locus 2 | |  | P97-RR1 | |  | P97-RR2 | |
| --- | --- | --- | --- | --- | --- | --- | --- | --- | --- | --- | --- |
| VNTR type | bp | repeats |  | bp | repeats |  | bp | repeats |  | bp | repeats |
| 1 | 181 | 6 |  | 108 | 4 |  | 335 | 12 |  | 314 | 4 |
| 2 | 145 | 4 |  | 96 | 3 |  | 335 | 12 |  | 284 | 3 |
| 3 | 127 | 3 |  | 96 | 3 |  | 305 | 10 |  | 344 | 5 |
| 4 | 217 | 8 |  | 84 | 2 |  | 320 | 11 |  | 314 | 4 |
| 5 | 235 | 9 |  | 96 | 3 |  | 350 | 13 |  | 254 | 2 |
| 6 | 235 | 9 |  | 84 | 2 |  | 245 | 6 |  | 284 | 3 |
| 7 | 253 | 10 |  | 84 | 2 |  | 245 | 6 |  | 284 | 3 |
| 8 | 271 | 11 |  | 84 | 2 |  | 245 | 6 |  | 284 | 3 |
| 9 | 289 | 12 |  | 84 | 2 |  | 245 | 6 |  | 284 | 3 |
| 10 | 163 | 5 |  | 96 | 3 |  | 290 | 9 |  | 344 | 5 |
| 11 | 163 | 5 |  | 96 | 3 |  | 320 | 11 |  | 344 | 5 |
| 12 | 163 | 5 |  | 96 | 3 |  | 275 | 8 |  | 374 | 6 |
| 13 | 145 | 4 |  | 96 | 3 |  | 275 | 8 |  | 374 | 6 |
| 14 | 145 | 4 |  | 84 | 2 |  | 335 | 12 |  | 344 | 5 |
| 15 | 145 | 4 |  | 84 | 2 |  | 320 | 11 |  | 314 | 4 |
| 16 | 181 | 6 |  | 84 | 2 |  | 320 | 11 |  | 314 | 4 |
| 17 | 181 | 6 |  | 84 | 2 |  | 335 | 12 |  | 344 | 5 |
| 18 | 199 | 7 |  | 84 | 2 |  | 335 | 12 |  | 344 | 5 |
| 19 | 217 | 8 |  | 84 | 2 |  | 290 | 9 |  | 344 | 5 |
| 20 | 235 | 9 |  | 84 | 2 |  | 290 | 9 |  | 344 | 5 |
| 21 | 235 | 9 |  | 84 | 2 |  | 290 | 9 |  | 284 | 3 |
| 22 | 253 | 10 |  | 84 | 2 |  | 290 | 9 |  | 344 | 5 |
| 23 | 271 | 11 |  | 84 | 2 |  | 290 | 9 |  | 344 | 5 |
| 24 | 289 | 12 |  | 84 | 2 |  | 290 | 9 |  | 344 | 5 |
| 25 | 307 | 13 |  | 84 | 2 |  | 245 | 6 |  | 344 | 5 |
| 26 | 163 | 5 |  | 84 | 2 |  | 320 | 11 |  | 314 | 4 |
| 27 | 163 | 5 |  | 84 | 2 |  | 335 | 12 |  | 344 | 5 |
| 28 | 127 | 3 |  | 120 | 5 |  | 380 | 15 |  | 344 | 5 |
| 29 | 181 | 6 |  | 96 | 3 |  | 275 | 8 |  | 374 | 6 |
| 30 | 145 | 4 |  | 96 | 3 |  | 305 | 10 |  | 374 | 6 |
| 31 | 109 | 2 |  | 84 | 2 |  | 350 | 13 |  | 374 | 6 |
| 32 | 109 | 2 |  | 96 | 3 |  | 335 | 12 |  | 374 | 6 |
| 33 | 235 | 9 |  | 84 | 2 |  | 320 | 11 |  | 374 | 6 |
| 34 | 109 | 2 |  | 84 | 2 |  | 335 | 12 |  | 374 | 6 |
| 35 | 127 | 3 |  | 96 | 3 |  | 275 | 8 |  | 374 | 6 |
| 36 | 127 | 3 |  | 96 | 3 |  | 275 | 8 |  | 404 | 7 |
| 37 | 109 | 2 |  | 72 | 1 |  | 320 | 11 |  | 344 | 5 |
| 38 | 217 | 8 |  | 84 | 2 |  | 305 | 10 |  | 344 | 5 |
| 39 | 127 | 3 |  | 84 | 2 |  | 290 | 9 |  | 374 | 6 |
| 40 | 127 | 3 |  | 96 | 3 |  | 290 | 9 |  | 374 | 6 |
| 41 | 127 | 3 |  | 84 | 2 |  | 290 | 9 |  | 374 | 6 |
| 42 | 199 | 7 |  | 72 | 1 |  | 305 | 10 |  | 344 | 5 |
| 43 | 109 | 2 |  | 72 | 1 |  | 305 | 10 |  | 344 | 5 |
| 44 | 199 | 7 |  | 72 | 1 |  | 290 | 9 |  | 344 | 5 |
| 45 | 127 | 3 |  | 84 | 2 |  | 350 | 13 |  | 284 | 3 |
| 46 | 127 | 3 |  | 84 | 2 |  | 335 | 12 |  | 284 | 3 |
| 47 | 127 | 3 |  | 84 | 2 |  | 320 | 11 |  | 284 | 3 |
| 48 | 145 | 4 |  | 96 | 3 |  | 350 | 13 |  | 284 | 3 |
| 49 | 145 | 4 |  | 96 | 3 |  | 230 | 5 |  | 284 | 3 |
| 50 | 217 | 8 |  | 84 | 2 |  | 335 | 12 |  | 344 | 5 |
| 51 | 127 | 3 |  | 96 | 3 |  | 380 | 15 |  | 344 | 5 |
| 52 | 127 | 3 |  | 120 | 5 |  | 275 | 8 |  | 344 | 5 |
| 53 | 127 | 3 |  | 96 | 3 |  | 245 | 6 |  | 344 | 5 |
| 54 | 127 | 3 |  | 96 | 3 |  | 275 | 8 |  | 344 | 5 |
| 55 | 127 | 3 |  | 96 | 3 |  | 290 | 9 |  | 344 | 5 |
| 56 | 199 | 7 |  | 96 | 3 |  | 275 | 8 |  | 374 | 6 |
| 57 | 163 | 5 |  | 96 | 3 |  | 335 | 12 |  | 374 | 6 |
| 58 | 163 | 5 |  | 96 | 3 |  | 275 | 8 |  | 314 | 4 |
| 59 | 163 | 5 |  | 96 | 3 |  | 320 | 11 |  | 374 | 6 |
| 60 | 163 | 5 |  | 96 | 3 |  | 230 | 5 |  | 314 | 4 |
| 61 | 163 | 5 |  | 96 | 3 |  | 260 | 7 |  | 314 | 4 |
| 62 | 145 | 4 |  | 96 | 3 |  | 275 | 8 |  | 344 | 5 |
| 63 | 253 | 10 |  | 84 | 2 |  | 245 | 6 |  | 344 | 5 |
| 64 | 145 | 4 |  | 84 | 2 |  | 245 | 6 |  | 344 | 5 |
| 65 | 289 | 12 |  | 96 | 3 |  | 290 | 9 |  | 284 | 3 |
| 66 | 397 | 18 |  | 96 | 3 |  | 290 | 9 |  | 284 | 3 |
| 67 | 397 | 18 |  | 96 | 3 |  | 305 | 10 |  | 284 | 3 |
| 68 | 505 | 24 |  | 96 | 3 |  | 290 | 9 |  | 284 | 3 |
| 69 | 523 | 25 |  | 96 | 3 |  | 290 | 9 |  | 284 | 3 |
| 70 | 523 | 25 |  | 96 | 3 |  | 305 | 10 |  | 284 | 3 |
| 71 | 505 | 24 |  | 96 | 3 |  | 305 | 10 |  | 284 | 3 |
| 72 | 289 | 12 |  | 96 | 3 |  | 305 | 10 |  | 284 | 3 |
| 73 | 145 | 4 |  | 96 | 3 |  | 290 | 9 |  | 374 | 6 |
| 74 | 163 | 5 |  | 96 | 3 |  | 290 | 9 |  | 374 | 6 |
| 75 | 109 | 2 |  | 96 | 3 |  | 305 | 10 |  | 344 | 5 |
| 76 | 109 | 2 |  | 96 | 3 |  | 335 | 12 |  | 344 | 5 |
| 77 | 109 | 2 |  | 84 | 2 |  | 305 | 10 |  | 374 | 6 |
| 78 | 109 | 2 |  | 96 | 3 |  | 305 | 10 |  | 374 | 6 |
| 79 | 163 | 5 |  | 96 | 3 |  | 290 | 9 |  | 434 | 8 |
| 80 | 163 | 5 |  | 96 | 3 |  | 305 | 10 |  | 434 | 8 |
| 81 | 163 | 5 |  | 108 | 4 |  | 290 | 9 |  | 374 | 6 |
| 82 | 217 | 8 |  | 84 | 2 |  | 305 | 10 |  | 374 | 6 |
| 83 | 235 | 9 |  | 84 | 2 |  | 290 | 9 |  | 374 | 6 |
| 84 | 145 | 4 |  | 108 | 4 |  | 290 | 9 |  | 374 | 6 |
| 85 | 217 | 8 |  | 96 | 3 |  | 290 | 9 |  | 344 | 5 |
| 86 | 217 | 8 |  | 96 | 3 |  | 305 | 10 |  | 344 | 5 |
| 87 | 145 | 4 |  | 96 | 3 |  | 245 | 6 |  | 434 | 8 |
| 88 | 145 | 4 |  | 96 | 3 |  | 275 | 8 |  | 434 | 8 |
| 89 | 163 | 5 |  | 96 | 3 |  | 260 | 7 |  | 374 | 6 |
| 90 | 163 | 5 |  | 96 | 3 |  | 275 | 8 |  | 404 | 7 |
| 91 | 145 | 4 |  | 96 | 3 |  | 320 | 11 |  | 284 | 3 |
| 92 | 163 | 5 |  | 96 | 3 |  | 275 | 8 |  | 344 | 5 |
| 93 | 199 | 7 |  | 96 | 3 |  | 290 | 9 |  | 374 | 6 |
